# Filling of Mater-Bi with Nanoclays to Enhance the Biofilm Rigidity

**DOI:** 10.3390/jfb9040060

**Published:** 2018-10-21

**Authors:** Giuseppe Cavallaro, Giuseppe Lazzara, Lorenzo Lisuzzo, Stefana Milioto, Filippo Parisi

**Affiliations:** Dipartimento di Fisica e Chimica, Università degli Studi di Palermo, Viale delle Scienze, pad. 17, 90128 Palermo, Italy; giuseppe.lazzara@unipa.it (G.L.); lorenzo.lisuzzo@unipa.it (L.L.); stefana.milioto@unipa.it (S.M.); filippo.parisi@unipa.it (F.P.)

**Keywords:** halloysite, sepiolite, laponite, nanoclays, Mater-Bi, bio-nanocomposites, mechanical performance

## Abstract

We investigated the efficacy of several nanoclays (halloysite, sepiolite and laponite) as nanofillers for Mater-Bi, which is a commercial bioplastic extensively used within food packaging applications. The preparation of Mater-Bi/nanoclay nanocomposite films was easily achieved by means of the solvent casting method from dichloroethane. The prepared bio-nanocomposites were characterized by dynamic mechanical analysis (DMA) in order to explore the effect of the addition of the nanoclays on the mechanical behavior of the Mater-Bi-based films. Tensile tests found that filling Mater-Bi with halloysite induced the most significant improvement of the mechanical performances under traction force, while DMA measurements under the oscillatory regime showed that the polymer glass transition was not affected by the addition of the nanoclay. The tensile properties of the Mater-Bi/halloysite nanotube (HNT) films were competitive compared to those of traditional petroleum plastics in terms of the elastic modulus and stress at the breaking point. Both the mechanical response to the temperature and the tensile properties make the bio-nanocomposites appropriate for food packaging and smart coating purposes. Here, we report a preliminary study of the development of sustainable hybrid materials that could be employed in numerous industrial and technological applications within materials science and pharmaceutics.

## 1. Introduction

Recently, eco-compatible polymers have been extensively investigated as potential alternatives to traditional plastics for several purposes within the packaging [1,2], biotechnology [3,4,5] and engineering [6,7,8] fields. The industrial use of biopolymers can be limited by their low barrier properties [9], thermal instability [10] and moderate mechanical behavior [11]. The addition of inorganic fillers to the biopolymer matrix represents an efficient strategy to fabricate composite materials with improved performance, which are suitable for numerous technological applications [11]. Ruiz-Hitzky et al. [12] highlighted that the filling of the polymeric matrix with clay nanoparticles enhanced the thermal and mechanical characteristics of pure polymers. Generally, the polymer/filler interactions can be favored by the high surface/volume ratio of the nanoclays, promoting an improvement of the mesoscopic properties of the nanocomposites [13]. The filling of polymers can be carried out using nanoclays with a variable shape, such sepiolite nanofibers [14], laponite nanodisks [15,16] and halloysite nanotubes [17,18]. The barrier properties of polylactic acid (PLA)-based films were improved by the addition of halloysite nanotubes (HNTs), allowing us to obtain hybrid materials useful for packaging [9]. The performance of the films was strongly dependent on the mesoscopic structure of the nanocomposites [18,19]. As a general consideration, the uniform distribution of the nanoparticles within the matrix determined the thermal stabilization of the polymers, because the filler acted as a barrier towards the volatile products generated by the polymer degradation [18,20]. Additionally, nanocomposites with a homogeneous morphology possess an improved mechanical resistance to tensile stress as a consequence of the adhesion of the polymers to the filler surface [21]. The literature [22,23] reports that composite films with a multilayer structure present peculiar characteristics. Flame retardant action was detected in multilayer nanocomposites formed by a middle clay layer sandwiched between the polymer [22]. As an example, a middle layer of montmorillonite between the alginate allowed the fabrication of multilayer bio-nanocomposite films with fire-shielding properties [16]. Recently, we proposed a sequential casting procedure to prepare flame-retardant films obtained by the confinement of HNTs between chitosan layers [22]. Among the nanoclays, halloysite represents an emerging filler with excellent properties in terms of the morphology and surface properties [24,25]. The peculiar tubular shape of halloysite is due to the rolling of flat kaolinite sheets [26,27]. The length of the HNTs ranged from 50 to 1500 nm, while the external and internal diameters ranged from 20–150 and 10–15 nm, respectively [27]. It should be noted that the polydispersity of the HNT sizes is affected by their geological deposit, as evidenced by microscopies [28] and neutron scattering [29] investigations. Interestingly, the halloysite surfaces exhibited opposite charges in a wide pH range (between 2 and 8) that can be attributed to their different chemistry, being that the shell and the lumen are composed of SiO_2_ and Al_2_O_3_ groups, respectively [30]. As proved by both in vitro and in vivo tests [31,32,33,34], HNTs can be considered to be biocompatible nanomaterials with a low toxicity effect. Accordingly, halloysite is suitable for biomedical and pharmaceutical applications as a nanocarrier for the controlled delivery of drugs [35,36,37]. HNTs have been successfully used as reinforcing nanofillers for several biopolymers, such as chitosan [22,38,39], cellulose ethers [18], pectin [17,21,40] and alginate [18]. The different HNT surface charge influences the properties of the bio-nanocomposites due to the specific electrostatic interactions occurring between ionic biopolymers and halloysite interfaces [18]. Anionic biopolymers are thermally stabilized due to their encapsulation within the HNT cavity as has been observed for nanocomposites based on alginate [18] and pectin [21]. Contrary to these results, the thermal stabilization effect was not observed for chitosan/HNT hybrid films where the biopolymer was adsorbed onto the halloysite external surface [18]. Sepiolite (Si_12_Mg_8_O_30_(OH)_4_(OH_2_)_4_·8H_2_O presents a nanofiber morphology with an average length between 1 and 2 µm and a diameter in the nanometric range (20–30 nm) [14]. Sepiolite nanofibers were used as nanofillers for poly (methyl methacrylate), improving both the thermal stability and the mechanical performance of the polymer [41]. Laponite (Si_8_(Mg_5.45_Li_0.4_)O_20_(OH)_4_Na_0.7_) possesses a disk-like shape with a diameter of ca. 25 nm and a thickness of 1 nm. Laponite nanodisks were filled with pectins, generating biofilms with moderate tensile properties [16]. Recently, nanocomposites based on poly (ethylene glycol) (PEG) silane and laponite were investigated as transparent non-fouling surfaces [42]. In this communication, we report the preparation and mechanical characterization of Mater Bi/nanoclay nanocomposite films with variable filler contents. Sepiolite nanofibers, laponite nanodisks and halloysite nanotubes were selected as the inorganic nanofillers. Dynamic mechanical analysis (DMA) provided the tensile properties as well as the thermo-mechanical behavior of the prepared films. The experimental data showed that filling Mater-Bi with halloysite allowed the fabrication of biocomposite films with promising mechanical performance for food packaging applications.

## 2. Results and Discussion

### 2.1. Tensile Properties of Mater-Bi/Nanoclay Composite Film

**Figure 1:**
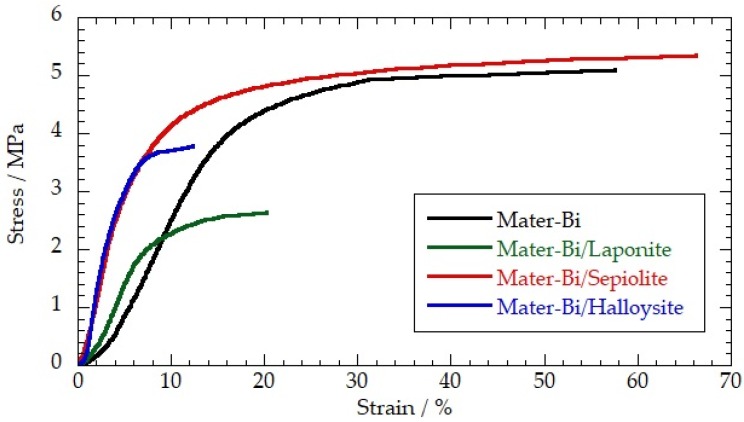
Stress–strain curves of Mater-Bi, Mater-Bi/ halloysite nanotubes (HNTs), Mater-Bi/sepiolite and Mater-Bi/laponite. The nanoclay content for the bio-nanocomposites was fixed at 30 wt %.

Figure 1 shows the stress–strain curves of pure Mater-Bi and the bio-nanocomposites with a nanoclay content of 30 wt %.

The analysis of the stress–strain profiles allowed us to determine a complete description of the tensile behavior of the prepared biofilms in terms of the elastic modulus, yielding and breaking points. The tensile data were collected in Table 1.

According to the literature results for the pectin/nanoclay nanocomposites [16], we observed that the nanofiller morphology affected the tensile characteristics of the hybrid biofilms. As a general result, the nanoclay addition generated an improvement in the film rigidity, as evidenced by the variations of the elastic modulus. This effect was significant for the Mater-Bi/HNT and Mater-Bi/sepiolite films, which showed relevant increases in the elastic modulus compared to that of the pure polymer (240% and 146%, respectively). On the other hand, the influence of the laponite nanodisks was negligible. With regard to the yielding point, the addition of the nanoclays reduced both the stress and elongation. The presence of the HNTs and laponite caused a decrease in the ultimate tensile strength as well as the maximum elongation. The latter can be attributed to the adsorption of Mater-Bi onto the nanoclay surface that avoids the sliding of the polymeric chains against each other [15,19]. Contrary to these results, the presence of sepiolite nanofibers did not alter the breaking point of Mater-Bi.

### 2.2. Thermo-Mechanical Beahaviour of Mater-Bi/Nanoclay Bio-Nanocomposites

**Figure 2:**
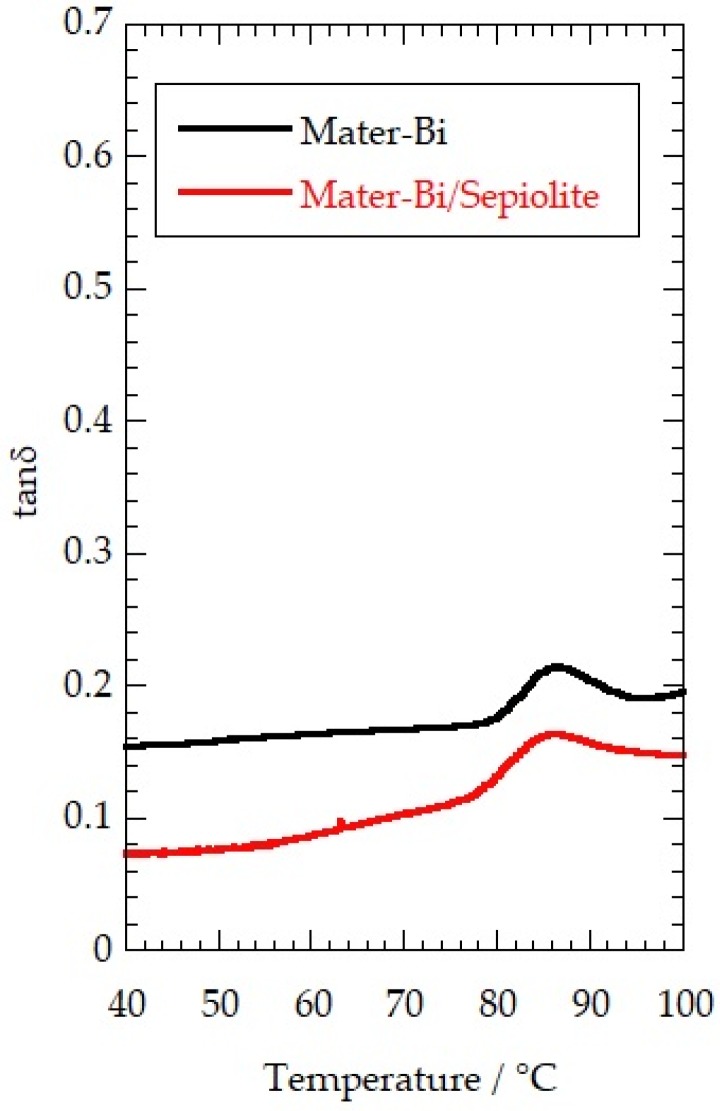
Dependence of tanδ on the temperature of Mater-Bi and Mater-Bi/sepiolite. The nanoclay content of the bio-nanocomposite was fixed at 30 wt %.

The mechanical response to temperature of the Mater-Bi based films was investigated by DMA test in the oscillatory regime. The obtained data allowed us to determine the effect of the temperature on the storage (G′) and loss (G″) moduli, which describe the viscoelastic characteristics of the materials. Figure 2 compares the dependence of tan (G″/G′ ratio) on the temperature of the Mater-Bi and Mater-Bi/sepiolite nanocomposite. We observed that tan exhibited a peak at ca. 85 °C due to the glass transition of the polymer. Similarly to sepiolite, the addition of HNTs and laponite did not change the glass transition temperature of Mater-Bi. Based on these results, we concluded that filling with nanoclays of variable shape does not alter the thermo-mechanical behaviour of the Mater-Bi biofilm.

## 3. Materials and Methods

### 3.1. Materials

The halloysite and 1,2-Dichloroethane were from Sigma-Aldrich (St. Louis, MO, USA). The sepiolite and laponite were from TOLSA S.A (Madrid, Spain) and BASF AG (Ludwigshafen, Germany), respectively. The Mater-Bi was a Novamont product (Novara, Italy).

### 3.2. Preparation of Mater-Bi/Nanoclay Nanocomposites

The solvent casting method for 1,2-Dichloroethane was employed for the preparation of the nanocomposites. Firstly, we prepared a 2 wt % Mater-Bi solution in 1,2-Dichloroethane by magnetically stirring for 2 h at 25 °C. Then, we added appropriate amounts of the nanoclay and the obtained dispersions were stirred overnight at 25 °C. The Mater-Bi/nanoclay mixtures were poured into glass Petri dishes at 25 °C to evaporate the 1,2-Dichloroethane. The obtained films were removed from the supports and stored in a desiccator at 25 °C. We selected 30 wt % as the filler concentration, which corresponds to the grams of nanoclay per 100 g of nanocomposite.

### 3.3. Methods

#### Dynamic Mechanical Analysis

DMA Q800 apparatus (TA Instruments, New Castle, DE, USA) was used to perform the dynamic mechanical analyses (DMA) of the Mater-Bi/nanoclay composite films. The DMA tests were conducted on rectangular films (10.00 × 6.00 × 0.060 mm^3^). Tensile investigations were carried out with a stress ramp of 1 MPa min^−1^ at 25.0 ± 0.5 °C. The mechanical response to the temperature was conducted in the oscillatory regime (with a frequency of 1.0 Hz and a strain amplitude of 0.5%) by heating the films from 40 to 100 °C at a heating rate of 5 °C min^−1^.

## 4. Conclusions

In summary, we prepared bio-nanocomposite films by filling Mater-Bi with several nanoclays using the solvent casting process. We observed that the presence of halloysite nanotubes and sepiolite nanofibers strongly increased the rigidity of the Mater-Bi-based films, extending the potential applications in the packaging fields. As evidenced by the variations of the elastic modulus, sepiolite and halloysite induced an increase of the Mater-Bi rigidity of 146% and 240%, respectively. The mechanical performance (regarding the yielding and breaking points) was still competitive with that of traditional plastics. The addition of the nanoclays did not affect the viscoelastic properties of the Mater-Bi and the polymer response to the temperature variations. In particular, the glass transition temperatures of the bio-nanocomposites were similar to that of the pure polymer.

## Figures and Tables

**Table 1:** Tensile properties of Mater-Bi and Mater-Bi/nanoclay films.

Film	Elastic Modulus/MPa	Stress at Yielding Point/MPa	Elongation at Yielding Point/%	Stress at Breaking Point/MPa	Elongation at Breaking Point/%
Mater-Bi	32 ± 3	3.8 ± 0.5	15.4 ± 1.8	5.0 ± 0.6	57 ± 6
Mater-Bi/HNTs	109 ± 8	2.8 ± 0.4	4.6 ± 0.5	3.8 ± 0.5	12 ± 2
Mater-Bi/Sepiolite	79 ± 7	3.4 ± 0.4	6.3 ± 0.6	5.3 ± 0.6	63 ± 6
Mater-Bi/Laponite	38 ± 4	1.98 ± 0.19	7.1 ± 0.6	2.6 ± 0.4	20 ± 2
